# Highlighting the Anti-Inflammatory Activity of Topical Gels Loaded with Meloxicam: The Role of Different Excipients and Absorption Promoters

**DOI:** 10.3390/polym18172065

**Published:** 2026-08-25

**Authors:** Ioana-Alexandra Plugariu, Luiza-Madalina Gradinaru, Irina Popescu, Maria Bercea

**Affiliations:** “Petru Poni” Institute of Macromolecular Chemistry, 41-A Grigore Ghica Voda Alley, 700487 Iasi, Romania; gradinaru.luiza@icmpp.ro (L.-M.G.); ipopescu@icmpp.ro (I.P.); bercea@icmpp.ro (M.B.)

**Keywords:** thermosensitive gels, polyurethane, meloxicam, anti-inflammatory activity, essential oil, excipients

## Abstract

Thermosensitive polyurethane (PU) hydrogels were prepared and loaded with meloxicam (MX), a nonsteroidal anti-inflammatory drug (NSAID). The role of different excipients, i.e., eucalyptus essential oil as well as poly(vinylpyrrolidone), poly(ethylene glycol) and their mixture, was highlighted. The sol–gel transition induced by temperature increase was evidenced by rheological measurements in oscillatory shear conditions. The viscoelastic behavior was investigated at 37 °C in various shear conditions, evidencing the network strength and stability of formulations. The structural recovery was monitored after applying high strain values, and the self-healing ability was revealed. Depending on the excipient, the gel samples loaded with MX release between 60% and 90% of the active substance. It was shown that the Fickian and chain relaxation contributions compete during MX release from PU-based hydrogels. In vitro anti-inflammatory activity was evaluated and discussed.

## 1. Introduction

The prevalence of chronic inflammatory diseases is rising, but the use of oral medications is limited by their gastrointestinal side effects. As a result, it is essential to develop topical formulations that either target a specific area locally or enable long-term treatment. Meloxicam (MX) is a potent nonsteroidal anti-inflammatory drug (NSAID) that belongs to the oxicam group [1,2,3]. MX preferentially inhibits cyclooxygenase-2 isoform (COX-2), and it is used in human medicine for the treatment of degenerative joint diseases (such as arthritis or osteoarthritis, rheumatoid polyarthritis or ankylopoietic spondylitis), as well as in ocular therapies and other applications [3,4,5,6,7]. Due to its anti-inflammatory, analgesic and antipyretic activities, this drug is also used in veterinary medicine in the treatment of musculoskeletal disorders, acute respiratory infection, osteoarthritis, puerperal septicemia or mastitis [2,8]. The limited solubility and lipophilicity of MX in most solvents hinder its incorporation into the majority of topical preparations [1,2,3,4]. There are currently no commercial topical MX products for human use authorized by the European Medicines Agency or found in standard European pharmaceutical databases. Clinical use is limited to oral (tablets, suspensions) or injectable routes [9,10,11]. However, in 2023, it was the 27th most commonly prescribed medication in the United States, with about 20.7 million total prescriptions [12], surpassing other NSAIDs, such as ibuprofen, acetylsalicylic acid or diclofenac. An intravenous version of meloxicam (Anjeso) was recently approved for medical use in the United States [13].

Therefore, the main interest is to include the efficient anti-inflammatory molecules of MX in appropriate systems that ensure optimal bioavailability and enable cutaneous absorption. For example, liposomal systems or gels based on β-cyclodextrins can easily incorporate hydrophobic active substances and facilitate their crossing of the phospholipid bilayer [3,4]. Pharmacosomes, vesicular systems that improve the absorption of poorly soluble substances and their bioavailability at the site of action, chemically bind the active substance and release it at the site of action, increasing the therapeutic efficacy [14]. Also, nanoparticle-based systems promote absorption and dissolution and prevent degradation of the active substance [15]. Another effective way to improve the thermodynamics quality of water in MX dissolution is the use of organic cosolvents, such as dimethyl sulfoxide, dimethylformamide or methanol, within acceptable safety limits for pharmaceutical products [16,17,18].

Excipients are generally inactive ingredients, but they can play a major role in achieving stable dosage forms and effectiveness of formulations, making them suitable for patients. Mainly, the excipients increase the bioavailability of the active substance or facilitate its passage through the skin or stratum corneum. Moreover, they can modify the active substance’s release profile by achieving a prolonged release or by accelerating it [19,20,21,22,23]. In topical gel formulations, absorption promoters significantly enhance the therapeutic effect of the active substance or enable rapid or controlled release, depending on the targeted condition. The release kinetics of the active substance is correlated with the rheological properties and anti-inflammatory activity of the gels. Excipients in novel MX formulations (e.g., hydrogels, emulgels, or vesicular phospholipid gels) modify their viscoelastic parameters and slow down the MX release [24,25].

Polyurethanes are biocompatible and hemocompatible polymers able to support cell growth and to mimic the biological and physical characteristics of both soft and hard tissues [26,27,28]. By modifying the building blocks, these structures can be customized for targeted drug release, controlling the delivery of the therapeutic agents over extended periods [29,30,31,32]. The tunable properties of polyurethanes make them suitable for soft tissue implants, wound dressings, surgical adhesives, implants, and cardiovascular devices [33,34,35,36,37].

In this context, the present study is focused on the investigation of some optimal formulations for chronic inflammatory conditions. Amphiphilic polyurethane (PU) hydrogels offer a powerful crosslinker-free mechanism for sustained drug delivery in chronic inflammatory diseases. They represent promising matrices, obtained by a crosslinker-free method, that facilitate the incorporation of the active substance. The thermally induced gelation enables the formulation to be used in both topical and injectable pharmaceutical forms. Concentrated PU solutions self-assemble into micelles at low temperatures and, upon encountering body temperature, undergo a spontaneous sol–gel transition, acting as a drug depot at the pathological site.

## 2. Materials and Methods

### 2.1. Materials

**Scheme 1:**
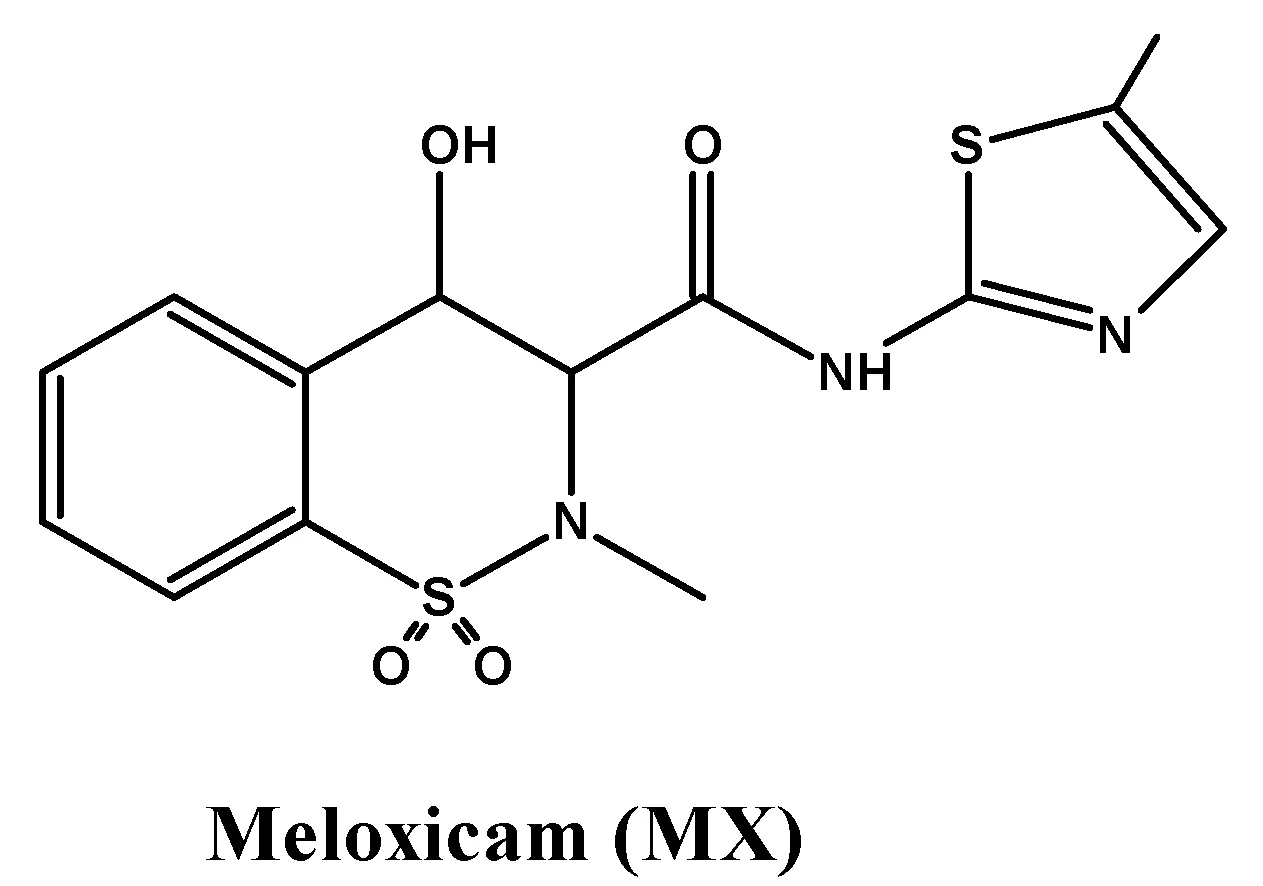
Molecular structure of meloxicam (MX), 4-hydroxy-2-methyl-N- (5-methyl-2-thiazolyl)-2H-1,2-benzothiazine-3-carboxamide-1,1-dioxide (C_14_H_13_N_3_O_4_S_2_).

The active substance used in this study, meloxicam, was purchased from Thermo Scientific (Leicestershire, UK). Its core chemical structure consists of a benzothiazine ring system coupled with an amide-linked thiazole ring (Scheme 1).

The NaOH used to dissolve meloxicam was obtained from Fluka Chemie GmbH (Buchs, Switzerland), and the NaCl also originated from the same source.

In the hydrogel formulations, various excipients were used, such as poly(vinylpyrrolidone) (PVP) with M = 4 × 10^4^ g/mol, which was purchased from Sigma Aldrich (Steinheim, Germany), poly(ethylene glycol) (PEG) with M = 4 × 10^2^ g/mol, and triethanolamine (TEA), purchased from Fluka Chemie GmbH (Buchs, Switzerland).

The eucalyptus essential oil was extracted from *Eucalyptus radiata* (family Myrtaceae) and supplied by Euflora (Iasi, Romania). The chemical composition is represented by eucalyptol (69.58%) and alpha-terpineol (10.32%).

To obtain the polymer matrix, the following chemicals were used: L-lysine ethyl ester diisocyanate, 97% (LDI), purchased from Alfa Aesar (Kandel, Germany), Pluronic P123, i.e., poly(ethylene oxide)_20_–*b*–poly(propylene oxide)_70_–*b*–poly(ethylene oxide)_20_ (PEO–*b*–PPO–*b*–PEO), M_n_ = 5.8 × 10^3^ g/mol, purchased from Sigma Aldrich (Steinheim, Germany), as well as 1,4-butane-diol (BD) as a chain extender.

### 2.2. Synthesis of Polyurethane Sample

**Scheme 2:**
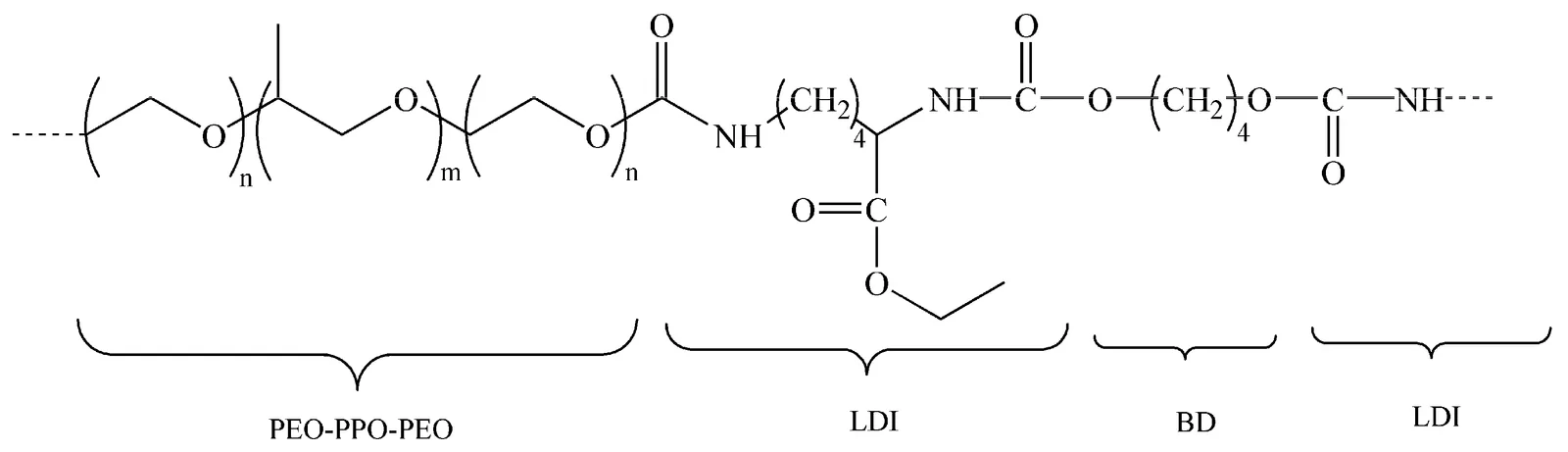
Structure of water-soluble polyurethane with thermosensitive properties [24].

The water-soluble polyurethane (PU) sample was synthesized via a standard two-step prepolymer method, as presented recently [24]. Firstly, the Pluronic (PEO–*b*–PPO–*b*–PEO) sample was dehydrated and then submitted to reaction at 80 °C for 4 h with an excess of LDI, without any catalyst, to obtain a prepolymer. Then, the BD was added and left for 6 h, under mixing. The reaction was carried out for a NCO:OH molar ratio of 1:1. The resulting PU structure is presented in Scheme 2.

### 2.3. Preparation of Thermosensitive Hydrogels

The first step in preparing the hydrogels was to dissolve the active substance, meloxicam, in a 0.5% aqueous NaOH solution under continuous stirring. All excipients were gradually added according to each formulation (Table 1). The samples were stirred throughout, and the polymer was added at the end. The samples were periodically alternated between room temperature and the refrigerator to accelerate polymer dissolution. The resulting hydrogel was homogenized using a rotary mixer. The pH values of final formulations were in the 7.5–8 range, which is acceptable for topical preparations [38]. This is primarily due to the presence of triethanolamine in the composition, which facilitates the solubilization of meloxicam, but TEA slightly increases the pH value. The PU-based hydrogels were stored at room temperature, where they remained stable for a longer period of time, without undergoing structural changes.

### 2.4. Characterization Methods

#### 2.4.1. Rheological Investigations

Rheological investigations were carried out using an MCR 302 Anton-Paar rheometer (Graz, Austria) equipped with a plane–plane geometry of 50 mm (with a gap of 500 µm). The temperature was rigorously controlled by using a Peltier device that permitted a fast temperature change. A device that creates a saturated solvent atmosphere was used to limit the water evaporation.

The gelation induced by temperature was investigated in an oscillatory shear regime with a heating rate of 1 °C/min, in the temperature range of 5 °C to 50 °C. The elastic (G′) and viscous (G″) moduli as well as the loss tangent (tan δ) were followed. G′ and G″ are correlated with the stored and dissipated energy during one cycle of deformation, respectively. The loss tangent (tan δ), as the G″/G′ ratio, is correlated with the sample’s viscoelasticity: tan δ < 1 reflects solid-like behavior, and tan δ > 1 indicates liquid-like behavior.

The gelation was investigated at the physiological temperature of 37 °C using solutions thermostated at 5 °C. During the experiment, the temperature was firstly set at 5 °C for 120 s, and then instantly changed to 37 °C. The viscoelastic parameters G′, G″ and tan δ were registered as a function of time at an oscillation frequency (ω) of 1 rad/s and strain amplitude (γ) of 1%.

The gel samples thermostated at 37 °C were studied in various shear conditions. The shear viscosity was determined at shear rates ($\dot{\gamma }$) from 0.01 s^−1^ to 1000 s^−1^. Amplitude sweep measurements allowed us to establish the linear viscoelastic regime and to determine the upper limit of strain (γ_L_) and the yield stress value (*τ*_o_). The thixotropy tests were followed in the oscillatory range for ω = 10 rad/s, and three step strains changed every 300 s from low (1% from the linear viscoelastic range) to high values (50%, 100%, 200%, 400%, 500%, 600%, 800% and 1000%—in the nonlinear domain of viscoelasticity) and again to the low step of strain (1%). Rheological measurements were performed in duplicate, and the experimental errors did not exceed 3%.

#### 2.4.2. Meloxicam Release from Hydrogels

We loaded 1% MX into the sol state, and the active molecules remained well dispersed in gel formulations. The release of the active substance was carried out using the dialysis method. In this step, 0.5 g hydrogel sample loaded with 1% MX was introduced into a dialysis membrane (with a cut-off molecular weight of 10 kDa). This membrane including the sample was placed in a closed glass container containing 15 mL of saline buffer solution (pH = 6) and placed in a thermostatic chamber at 37 °C. Here, 1 mL from the release medium was collected at different time intervals. Simultaneously, 1 mL of saline buffer solution thermostated to the same temperature was added in order to maintain a constant volume of the release medium. The amount of delivered meloxicam was determined spectrophotometrically using a calibration curve for MX. The absorbance was recorded at 363 nm with a Libra UV-Vis spectrophotometer (Biochrom Libra S35PC, Cambridge, UK), using MX solutions with concentrations in the range 0.0030–0.0102 mg/mL. The experimental investigation was performed in triplicate.

#### 2.4.3. Evaluation of Anti-Inflammatory Effect of the Hydrogel Samples

The anti-inflammatory activity of the hydrogel samples was validated by their ability to inhibit the bovine serum albumin’s denaturation [39]. An in vitro test was conducted using a 5% bovine serum albumin solution, adjusted to an optimal pH of 5.5.

A quantity of 0.6 g of the hydrogel sample with different compositions was taken, and over 6 mL of 5% BSA was added. The final solution was brought at a pH of 5.5 by using 0.1 N hydrochloric acid.

The control sample was prepared by replacing the amount of hydrogel with distilled water. Here, 3 mL of each mixture was taken, and the samples were incubated at room temperature for 20 min. Subsequently, the samples were thermostated at 70 °C for 5 min. The appearance of opalescence was considered an indicator of protein denaturation.

After the heating process, the control and the samples were allowed to cool, and absorbance was measured at 660 nm using a UV-Vis spectrophotometer. Diclofenac sodium was used as the standard active substance for the prepared samples. The sample containing the BSA denatured solution served as the control. The experimental study was performed in triplicate.

The degree of protein inhibition can be determined using the following equation:

(1) $$Inhibition\;(\%)=\frac{A_{control}-A_{sample}}{A_{control}}\times 100$$

where *A_control_* and *A_sample_* are the absorbance values of the control and the hydrogel sample.

This equation shows the percentage of inhibition of protein denaturation for each sample, with values directly proportional to the anti-inflammatory activity.

### 2.5. Statistical Analysis

OriginPro 8.5 was used to process the experimental data concerning MX release, and the minimum residual sum of squares (RSS) was considered [40]. The Akaike Information Criterion (AIC) was used as a discriminatory criterion, independent of the number of parameters in each model [41]:

(2) AIC=Nln(RSS)+2p

where *p* represents the number of parameters involved in a given model and *N* is the number of experimental data used in evaluation. The model that best describes the release kinetics is the one with the lowest AIC value.

## 3. Results

### 3.1. Rheology of Hydrogels

#### 3.1.1. Temperature-Induced Gelation

**Figure 1:**
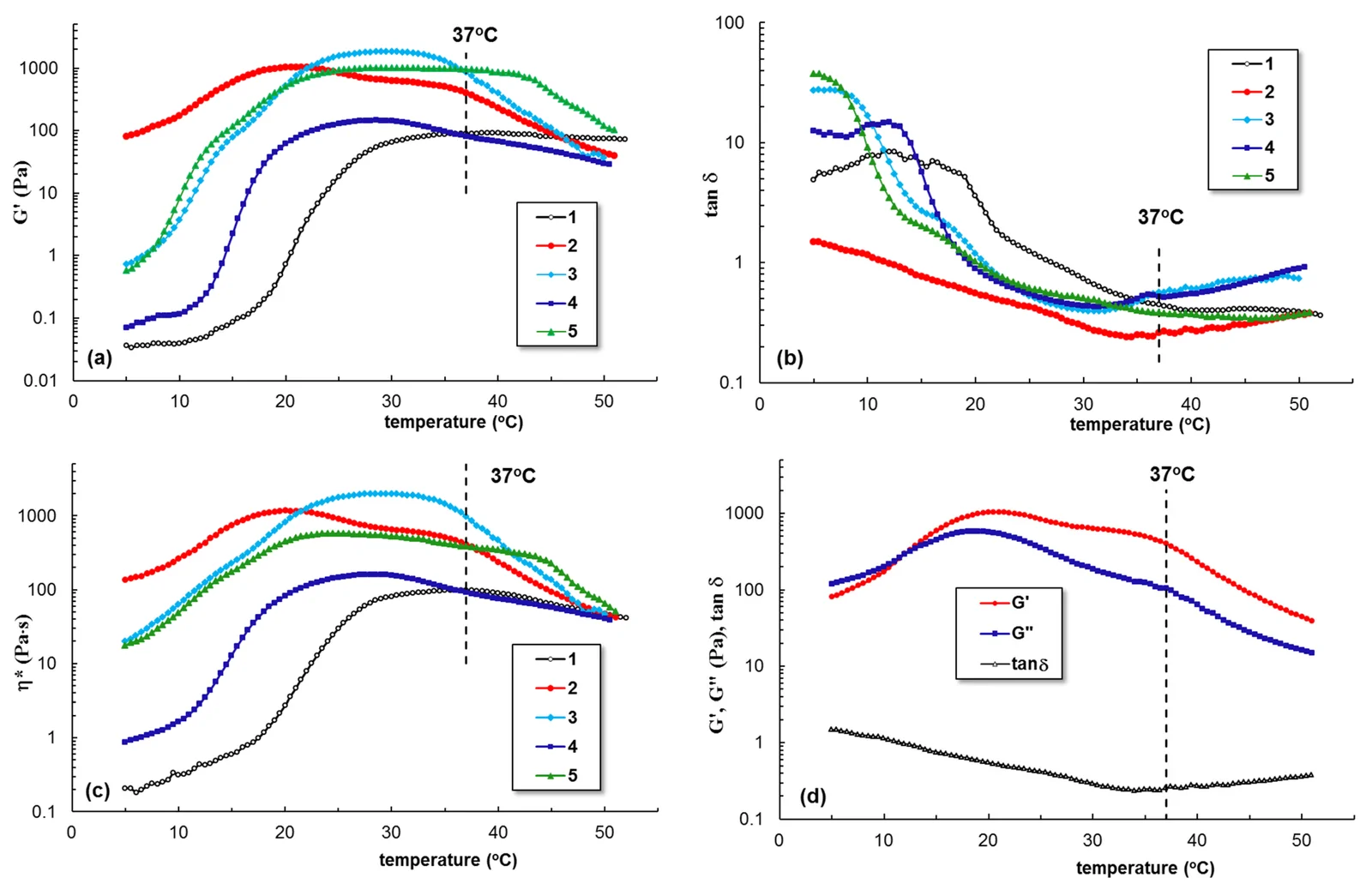
The evolution of viscoelastic parameters as the temperature increases with 1 °C/min: (**a**) elastic modulus; (**b**) loss tangent; (**c**) complex viscosity and (**d**) G’, G” and tan δ for sample 2 (Table 1).

At low temperatures, PU dissolved in a water/TEA mixture, incorporating MX, behaves as a viscous fluid (Figure 1). In aqueous media, nanomicelles with a hydrophobic core and a hydrophilic shell are formed by self-assembly of PU chains [42,43]. In this state, it was easier to incorporate the hydrophobic MX and the other excipients (samples 2–5, Table 1). At an increasing temperature, a sol–gel transition occurs, accompanied by a sharp variation in the viscoelastic parameters (Figure 1a–c).

Each component added to the system influences the behavior during micellization and gelation. The eucalyptus essential oil stabilizes the sample at low temperatures and increases the values of G′, G″ and η*. The essential oil is lipophilic, and when it is mixed with the polymer above the critical micelle concentration of PU, the essential oil molecules are encapsulated inside the micelle core, while the hydrophilic heads face outward. This significantly boosts the stability, water solubility, and functionality of the essential oil in the formulation and influences the viscoelasticity of the sample (above 15 °C, G′ > G″ and tan δ < 1, Figure 1d). The addition of the long PVP chains also changes the viscoelasticity of PU due to the hydrogen bonding and chain entanglements. The short PEG chains form hydrogen bonds with PU micelles, increasing the viscosity and viscoelastic moduli at low temperatures, but this increase is smaller than that observed by PVP addition. Above 35 °C, these interactions are weakened by thermal effect. Sample 5 contains both PVP and PEG and exhibits a combined behavior of the two polymers.

#### 3.1.2. Viscoelastic Behavior at 37 °C

**Figure 2:**
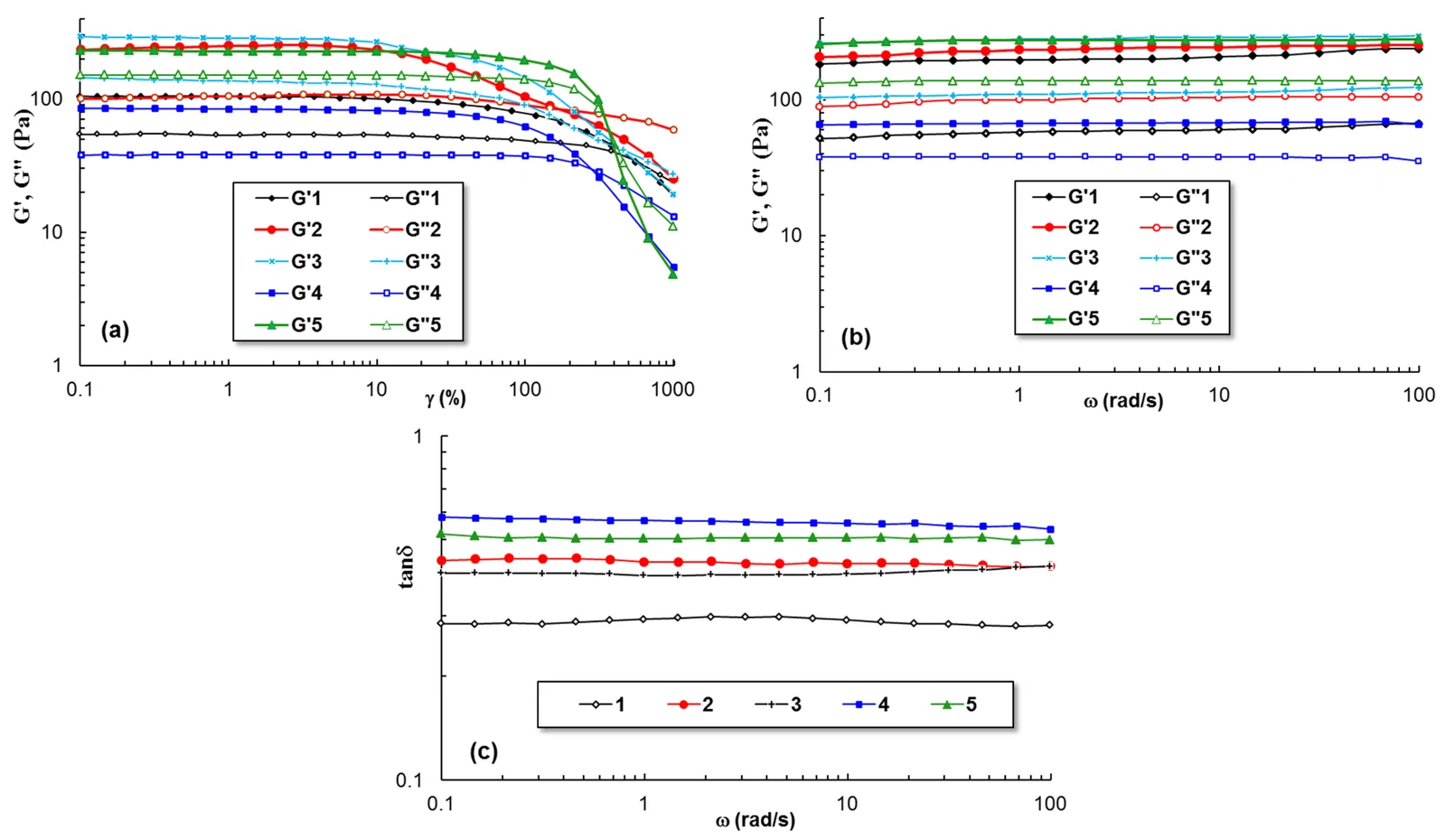
The viscoelastic moduli (G′ and G″) as a function of (**a**) strain amplitude (ω = 1 rad/s), (**b**) oscillation frequency (γ = 1%) and (**c**) the loss tangent in frequency sweep test for hydrogels thermostated at 37 °C.

All samples thermostated for 24 h at 37 °C are in a gel state, with G′ > G″ and tan δ < 1 (Figure 2). In amplitude sweep tests, the linear viscoelastic range (the domain in which a linear relationship between stress and strain is registered) was delimited at low strain (γ) values (Figure 2a). The nonlinear viscoelastic range is registered above 10% for sample 2, whereas for sample 1 (PU) and samples 3–5 (containing polymer excipients), the linear range is reached at higher γ values (around 100%). Macromolecular chains respond slowly to applied deformation and extend the linear viscoelastic range by enabling the molecular rearrangements, disentanglements, and the breaking/reforming of physical bonds to dissipate stress. This prevents the rapid release of active substance incorporated into the network when the gel is submitted to deformation during the topical application.

In frequency sweep tests, the viscoelastic moduli (G′ and G″) are almost independent of oscillation frequency (Figure 2b), and the loss tangent is lower than unity. The presence of excipients reduces the degree of viscoelasticity in the polymicellar network of PU (tan δ increases for samples 2–4), which could be beneficial for MX release.

#### 3.1.3. Shear Behavior

**Figure 3:**
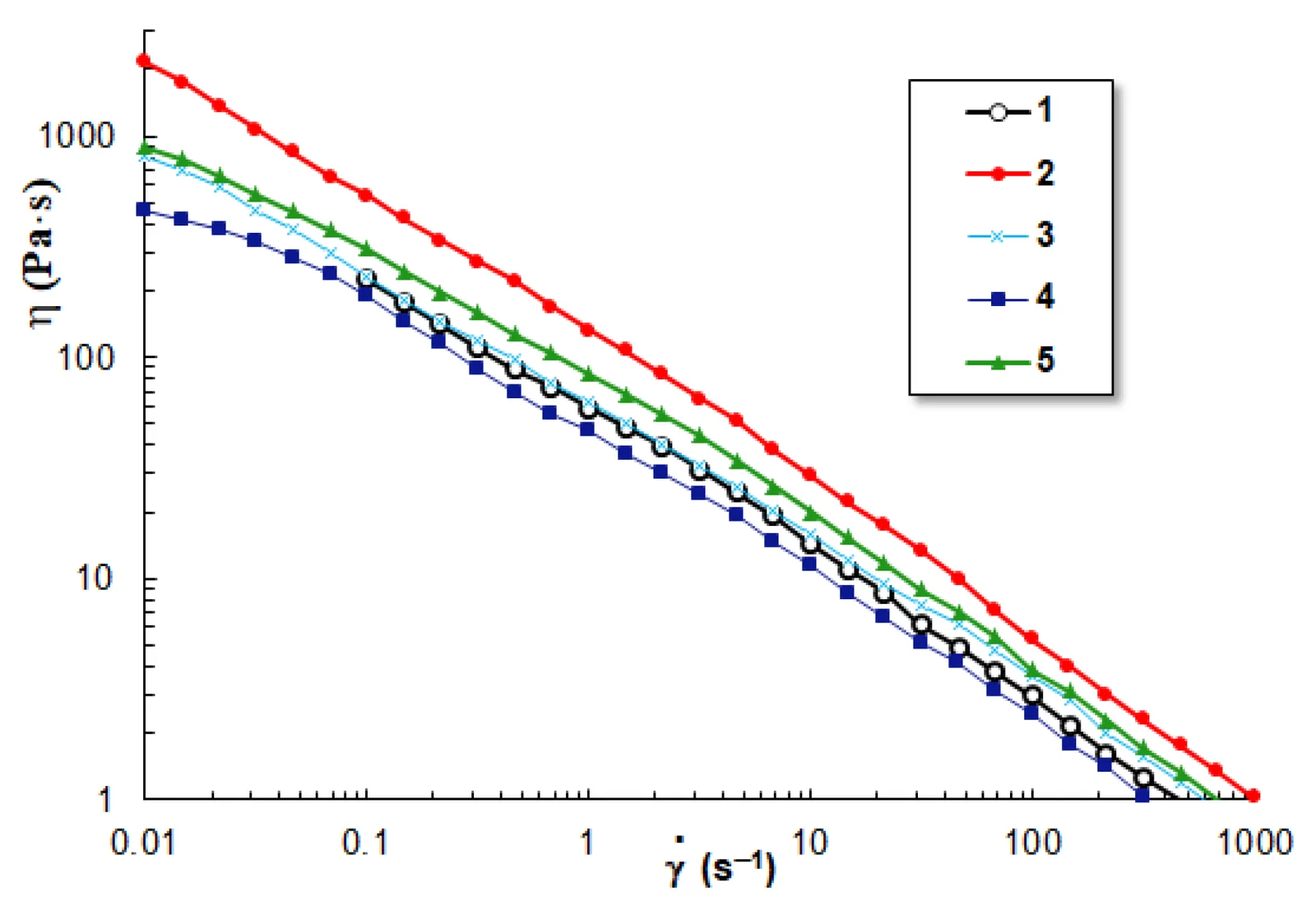
The shear viscosity as a function of shear rate for hydrogel samples at 37 °C.

Figure 3 shows the variation in apparent viscosity (*η*) as a function of the applied shear rate ($\dot{\gamma }$). Above the gelation temperature, the different structural entities present in the PU-based network (polymicelles, polymer chains, micelles, aggregates) display non-Newtonian flow under the action of the shear rate. The experimental data were fitted using the Ostwald-de-Waele model:

(3) $$\eta =K\;{\dot{\gamma }}^{n-1}\mathrm{or}\;\tau =K\;{\dot{\gamma }}^{n}$$

where *K* is the consistency coefficient (Pa × s*^n^*), *n* is the flow index and *τ* is the shear stress.

For the investigated PU-based hydrogels, the slope of *η* = f($\dot{\gamma }$) dependence was 0.65 ± 0.03, whereas the *K* values were 57.52, 130.22, 61.44, 45.68, and 81.13 Pa·s^0.65±0.03^ for samples 1–5, respectively. Thus, the flow index value is *n* = 1.65 ± 0.03, showing a strong non-Newtonian behavior.

Yield stress (*τ*_o_) is an important rheological parameter, representing the minimum shear stress required to break the internal structure and initiate the flow of the sample. Its determination is essential for understanding the mechanical behavior of different hydrogels, as the transition point at which the sample begins to change its structure from a solid-like (elastic) behavior to a liquid-like flow. For the hydrogel samples, *τ*_o_ values were determined in amplitude sweeps by tracking how the storage and loss moduli change as shear stress increases [44,45,46]. The yield stress is a determining factor in applications such as drug delivery, injectable scaffolds or 3D bioprinting [46].

For the PU hydrogels loaded with MX, the *τ*_o_ value varies with the sample composition (Table 1). PEG lowered the *τ*_o_ value, whereas PVP and eucalyptus essential oil increased the flow resistance. Below its yield stress, the gel acts like an elastic solid, stores energy internally and undergoes reversible deformations. When the stress or deforming force is removed, the gel snaps back to its original shape. For shear stress values exceeding *τ*_o_, the intermolecular interactions begin to break under the action of shear forces, and the sample begins to flow. The material transitions into a fluid state when the macromolecules tend to orient along the flow direction.

Shear forces cause a decrease in viscosity, which facilitates the application of the gel over a larger surface area and allows the drug molecules to diffuse into the skin layers [47].

#### 3.1.4. Self-Healing Behavior

**Figure 4:**
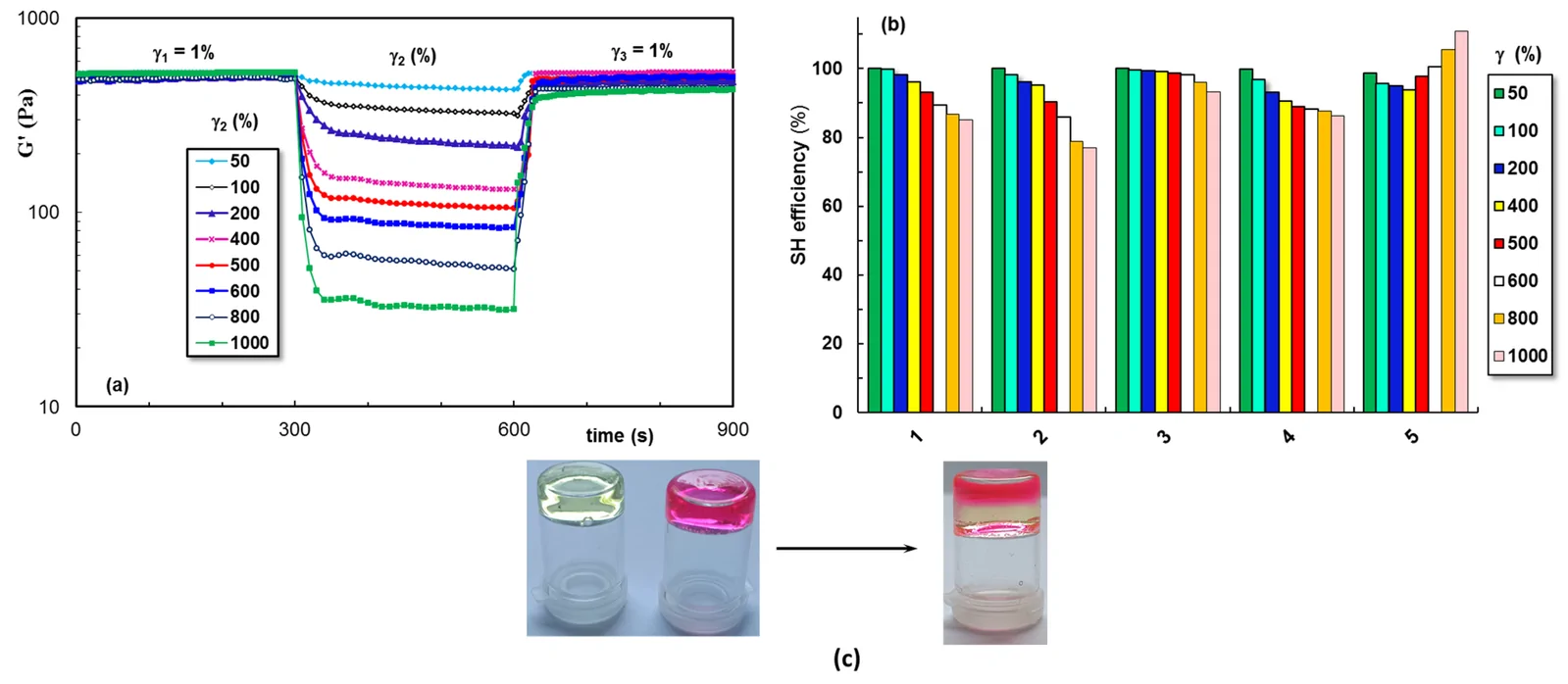
(**a**) Illustration of the self-healing ability for sample 2 submitted successively to various γ_2_ values; (**b**) self-healing efficiency for the investigated gel samples after applying three strain step cycles; (**c**) visualization of self-healing behavior: when two separated pieces of hydrogel are put in contact, they autonomously undergo a structural recovery in a few minutes due to reestablishment of hydrogen bonding and hydrophobic associations.

Thixotropy tests were carried out for all hydrogels loaded with MX, and the viscoelastic parameters were monitored as a function of time in order to investigate the extent of structure recovery. The dependences of G′, G″ and tan δ were examined for ω = 10 rad/s and three steps of strain applied successively: γ_1_ = 1% (which is in the linear domain of viscoelasticity), various high values of γ_2_ (in the nonlinear region of viscoelasticity) and then γ_3_ was set again to 1%. Figure 4a presents, as an example, the behavior of sample 2 in the thixotropy tests. For γ_2_ ≤ 400%, the initial values of elastic modulus registered during step 1 (G′_1_) are rapidly recovered to a very large extent during step 3 (G′_3_). By applying higher strain values (γ_2_ ≥ 500%), the gel’s structure is disturbed, and the recovered G′_3_ values are lowered as γ_2_ becomes greater.

In order to compare the different hydrogel formulations, the self-healing efficiency was defined as

(4) $$SH\;efficiency=\frac{{G^{\prime }}_{3}}{{G^{\prime }}_{1}}$$

where G′_3_ is the elastic modulus registered after applying a given value of γ_2_, and G′_1_ is the initial value of elastic modulus obtained during step 1.

According to Figure 4b, the SH efficiency decreases as γ_2_ increases in the case of sample 1; the decrease is more pronounced in the presence of eucalyptus essential oil (sample 2), similar to the behavior depicted in Figure 2a. SH efficiency is above 90% for the PU gels in the presence of polymers (PVP—sample 3 and PEG—sample 4). A synergistic effect is registered for sample 5, which contains both PEG and PVP, and the gel structure is strengthened under the action of high deformations. The PVP and PEG macromolecular coils unfold under the action of high deformation and then allow the system to reorganize into a stronger network in which PU micelles coexist with the unfolded macromolecules that interact with the micelle’s corona.

### 3.2. In Vitro Drug Release

It was shown that TEA stabilizes the MX-loaded hydrogel and enhances the transdermal delivery of the active substance. It forms a reversible ion pair with MX, increasing its solubility within the hydrogel [48]. In the presence of this absorption promoter, the MX release, bioavailability and therapeutic efficacy increase [49]. Thus, in all hydrogel formulations, 3% TEA was added (Table 1).

Furthermore, poly(ethylene glycol) (PEG) improves the hydrogel’s biocompatibility. Poly(vinylpyrrolidone) (PVP), added in small amounts in hydrogels, enhances the dissolution of the active substance, and combining it with PEG yields an optimized formulation. It was shown that 1,8-cineole, a component of eucalyptus essential oil, significantly enhances the therapeutic effect of the active substance or enables rapid or controlled release, stimulating the absorption of meloxicam at the site of action [50]. Another property of eucalyptus essential oil is its anti-inflammatory activity, with all formulations characterized by this important effect in the treatment of various pathologies [51]. Eucalyptus essential oil accelerates the release of the active substance, thereby acting as an absorption promoter [52].

**Figure 5:**
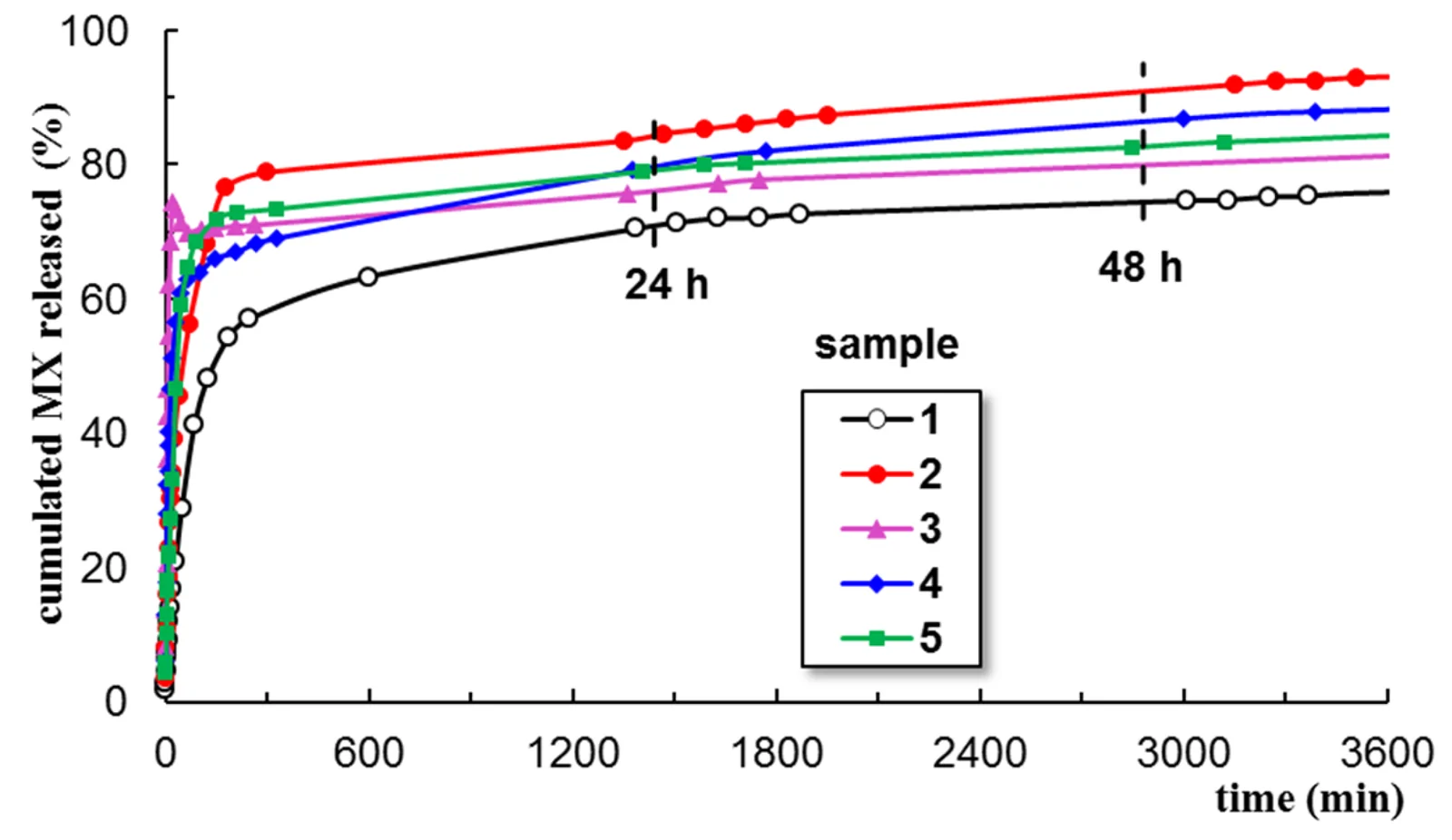
Cumulative MX release from PU-based hydrogels (37 °C).

The influence of different excipients on the delivery profile of MX from the formulations was monitored, and the results are presented in Figure 5. The MX cumulative release from the PU gel is about 60% after the first 300 min, and then it increases slowly and reaches about 70% after a long period of time. On the other hand, sample 2, containing eucalyptus essential oil as an absorption promoter, with eucalyptol (1,8-cineole) as its main component, releases about 80% MX over the first 300 min. According to literature data [52], eucalyptus essential oil increases the diffusion of the active substance through the skin. The mechanism by which eucalyptus essential oil promotes the absorption of meloxicam involves direct action on the lipid layer and an improvement in the active substance’s partition coefficient [53]. Thus, the skin barrier permeability and the lipophilicity of meloxicam are improved, enhancing the release profile and anti-inflammatory potential of meloxicam. This excipient has the advantage of lower toxicity compared to synthetic absorption promoters [53]. It accelerates the wound healing and reduces inflammation. The key terpene compounds—particularly 1,8-cineole, α-terpineol, and limonene—display strong antibacterial, antifungal, and analgesic effects, inhibiting the biofilm formation and promoting collagen synthesis.

In addition to the basic PU formulation, sample 3 contains 3% PVP and presents an improved delivery profile. The presence of this polymer improves the gel’s spreadability, thereby achieving an optimal distribution of the active substance [54].

PEG 400 (introduced in sample 4) acts as a plasticizer, accelerating the release of the active ingredient [55,56]. The formulation containing PVP and PEG 400 (sample 5) yields stable hydrogels, as these compounds are completely miscible due to hydrogen bonding between them [57].

The kinetics of MX delivery from the various hydrogels were analyzed by using different models, and the results of these evaluations are given in Table 2. Two versatile models developed by Peppas and coworkers [58,59] have provided the best fit of the experimental data. *M_t_*/*M*_∞_ represents the fraction of the drug released at time *t*. The release constants, denoted generally by *k*, depend on the drug characteristics [58,59,60]. The equations are suitable up to 60% of the maximum level of drug release, and the exponents *m* and *n* are correlated with the release mechanism of the drug from the hydrogel (Fickian or non-Fickian diffusion, etc.). The constants *k*_1_ and *k*_2_ from Peppas and Sahlin’s model reflect the Fickian or non-Fickian contributions to diffusion during the drug delivery [59]. Low negative AIC values were obtained for most samples by applying the Peppas–Sahlin model. The exponents *m* and *n* are higher than 0.5 in the case of simple PU hydrogel, suggesting anomalous transport phenomena (non-Fickian diffusion). For the other PU-based hydrogels (samples 2–5), values of *n* < 0.5 were obtained (Table 2), reflecting a pseudo-Fickian diffusion with slow drug release [58,61,62]. The Higuchi model is more suitable for samples 1, 4 and 5, whereas the first-order approximation is more reliable for samples 1 and 5.

According to Peppas and Sahlin’s model [59], *m* is the purely Fickian diffusional exponent; *k*_1_ represents the kinetic constant for the Fickian diffusional contribution and *k*_2_ is the kinetic constant for the Case-II relaxation contribution. By separating these two distinct contributions (Fickian diffusion and Case-II relaxation), this dual-mechanism approach generalizes the delivery behavior and avoids the uncertainty of the classic single-exponent Korsmeyer–Peppas power law [58]. Thus, Peppas and Sahlin’s model is able to estimate which transport process governs a dissolution profile for better approximation of drug release with ongoing anomalous transport phenomena. By writing the released fraction as two additive power terms, the model allows us to determine, at any time point, whether the drug is released mainly by water-driven diffusion through swollen gel, by polymer chain relaxation, or by a coupling of both (anomalous, non-Fickian transport). This distinction is important when reformulating a controlled-release product or determining a profile that fluctuates between batches.

Each contribution (Fickian diffusion or relaxation transport) to the delivery profile can be discussed through the fractional Fickian (*F*) and relaxational (*R*) contributions [66,67,68]:

(9) $$F=\frac{1}{1+\frac{k_{2}}{k_{1}}\;t^{m}}$$

(10) $$R=\frac{\frac{k_{2}}{k_{1}}\;t^{m}}{1+\frac{k_{2}}{k_{1}}\;t^{m}}$$

Thus, for $\frac{k_{2}}{k_{1}}\;t^{m}\to 0$, the release is entirely governed by Fickian diffusion, and for $\frac{k_{2}}{k_{1}}\;t^{m}\to \infty$, the release is entirely governed by Case-II relaxation.

**Figure 6:**
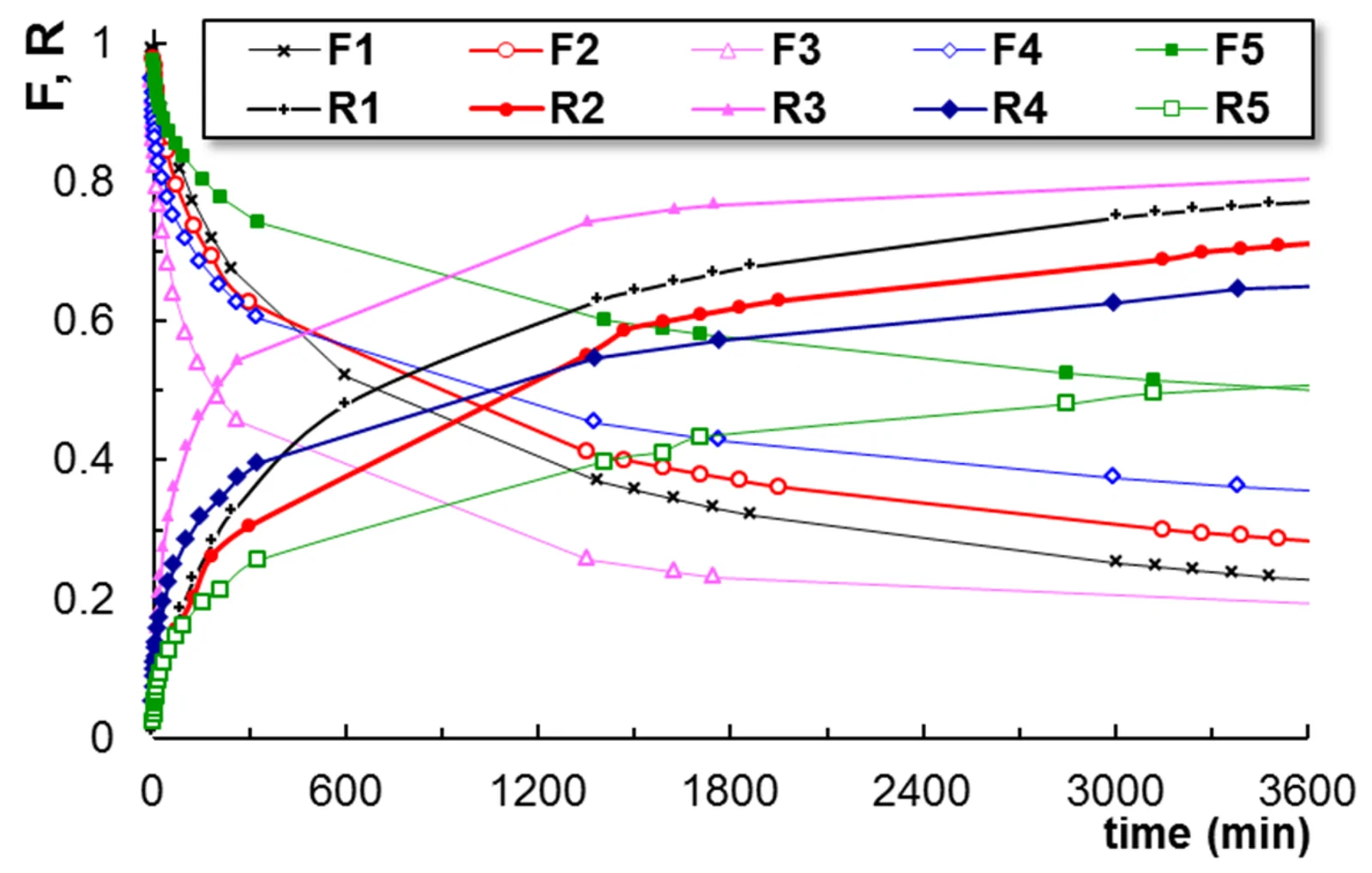
The contributions of Fickian diffusion (*F*) and relaxation (*R*) to MX release from the PU-based hydrogels.

According to Figure 6, the fractional Fickian (*F*) contribution prevails during the first release step, and then the relaxational (*R*) contribution dominates, both contributing to MX release. For sample 3, diffusion is predominant during the first 200 min, and then a faster increase in relaxation is observed. The slowest relaxation occurs for sample 5, when the diffusion is predominant during MX release for approx. 3300 s.

### 3.3. Anti-Inflammatory Activity

The inhibition of protein denaturation assay is an indirect in vitro method used to screen plant extracts, natural compounds, or synthetic drugs for anti-inflammatory activity [39,69,70,71,72]. Protein denaturation (often caused by heat or chemical stress) is accompanied by a loss of its tertiary structure, and consequently the functional properties are altered. The agents that prevent this change demonstrate potential therapeutic anti-inflammatory properties.

**Figure 7:**
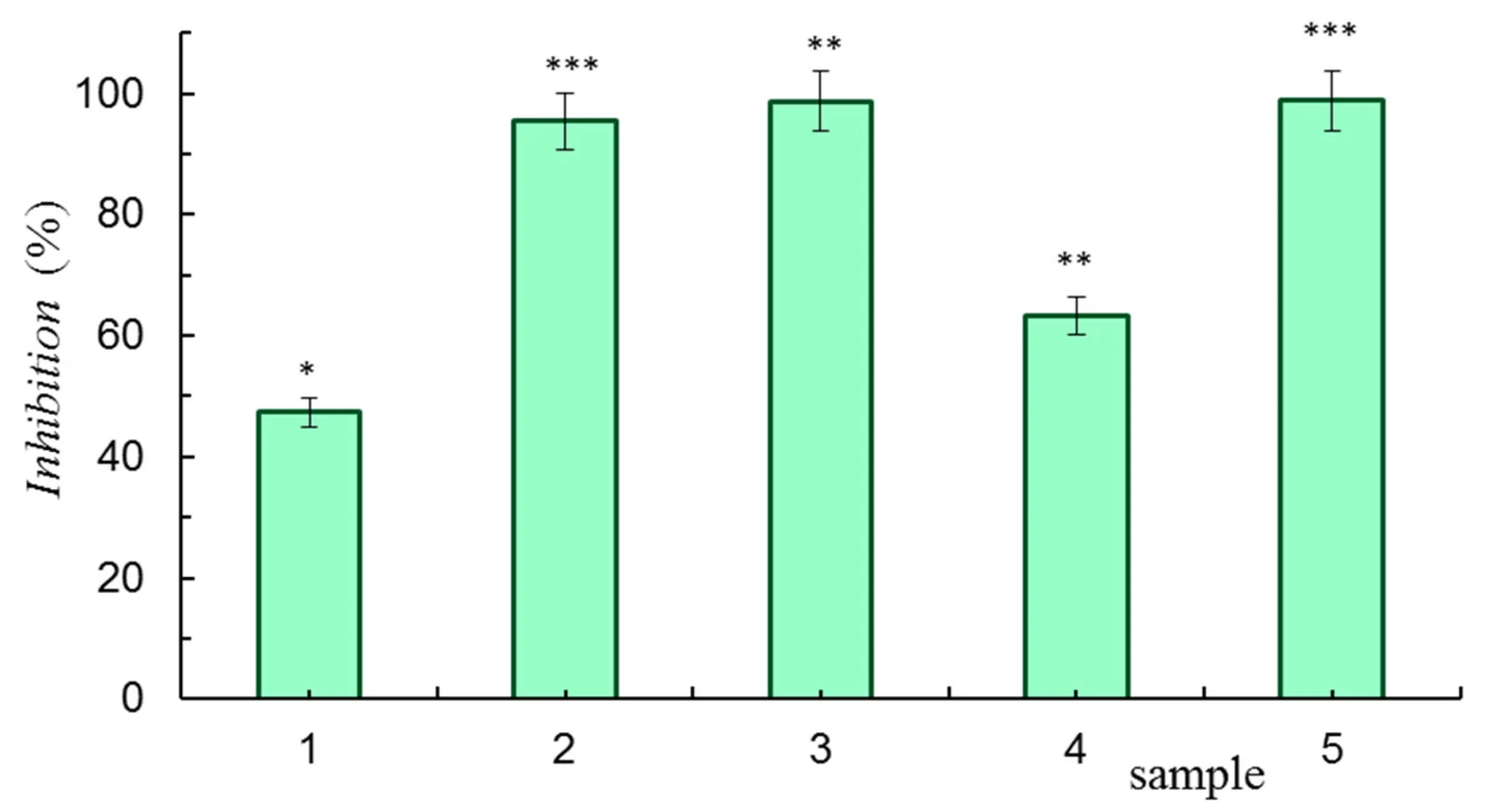
The BSA inhibition evaluated according to Equation (1) for PU-based hydrogels. Statistical significance: *** *p* < 0.001; ** 0.001 < *p* < 0.01; * 0.01 < *p* < 0.05.

In the present study, the anti-inflammatory effect of the hydrogels loaded with MX was assessed by their ability to inhibit the denaturation of bovine serum albumin (BSA). All samples demonstrated anti-inflammatory activity, with the effect varying with the chemical composition (Figure 7).

PU hydrogels loaded with MX showed improved physicochemical stability when combined with triethanolamine (TEA). Upon interaction between MX and TEA, a corresponding salt forms, increasing the solubility of poor water-soluble meloxicam molecules. TEA is also considered an absorption promoter by increasing the availability of the active substance at the site of action. Furthermore, TEA provides a basic pH, favorable to MX dissolution [49]. Therefore, all samples had, as their basic composition, a PU amphiphilic matrix favorable to incorporate poorly soluble substances, the anti-inflammatory active substance meloxicam at a therapeutically effective dose, and TEA. The difference in anti-inflammatory activity percentages was due to the varying excipients added to the samples. Thus, a difference in anti-inflammatory activity is observed between sample 2 (*Inhibition* = 95.43%) and sample 1 (*Inhibition* = 47.32%). Sample 2 additionally contains *Eucalyptus radiata* essential oil. This excipient is considered an absorption promoter, but is also used in formulations for anti-inflammatory and antiseptic properties [73]. 1,8-cineole, the active substance from eucalyptus essential oil, increases the availability of the active substance by modifying permeability. Thus, by acting on the lipid layer, it acts synergistically with MX, potentiating its therapeutic effect in various topical inflammatory conditions [50,51]. Li et al. have shown that a topical nanoemulsion gel containing 1,8-cineole did not cause skin irritation [74]. Furthermore, according to the European Medicines Agency monograph, eucalyptus essential oil may be used in semi-solid preparations at a concentration of up to 10% [75].

PVP added as an excipient in sample 3 stabilizes the gel by ensuring optimal viscosity (Figure 3). Also, PVP enhances the dissolution of MX, thereby increasing the bioavailability of the active substance and its anti-inflammatory effect [55,76].

In addition to the basic composition, sample 4 contains low-molecular-weight PEG as an excipient. This favors the dissolution of meloxicam with beneficial effects on the availability of the active substance at the site of action and its anti-inflammatory effect [77]. Unlike sample 4, sample 5 has shown a higher capability of protein denaturation inhibition (*Inhibition* = 98.81%) than sample 4 (*Inhibition* = 63.36%). The difference between these samples is represented by the association of PVP with PEG in the formulation. Both excipients (PVP and PEG) added to the PU-based formulation increase the solubility of the poorly soluble active substance and enhance wetting, leading to stable hydrogels that are absorbed more quickly. Thus, the active substance reaches the site of inflammation more quickly with a faster and more pronounced expression of the therapeutic effect [78,79,80]. PVP and PVP/PEG mixtures are biocompatible and suitable for topically administered hydrogels [81,82].

## 4. Conclusions

This paper presents new results concerning the use of the thermosensitive polyurethane hydrogels as a matrix for incorporating an anti-inflammatory compound, MX. The addition of eucalyptus essential oil, PVP, PEG and PVP/PEG mixture as excipients changes the rheology of the formulation and influences the MX delivery. The excipients induce tunable viscoelasticity and high self-healing ability, especially the PVP/PEG mixture.

The MX delivery is governed by non-Fickian diffusion for simple PU hydrogel and pseudo-Fickian diffusion with slow drug release for PU-based hydrogels, containing different excipients. It was found that diffusion was the predominant mechanism during the first 3 h, and then the contribution of chain relaxation becomes significant. Eucalyptus essential oil and PVP were identified as promising agents that potentiate the anti-inflammatory activity of PU gels loaded with MX.

## Figures and Tables

**Table 1:** Composition of the hydrogel samples, yield stress (*τ*_o_) and viscoelastic parameters determined at 37 °C.

Sample Code	PU (%)	TEA (%)	MX (%)	Excipient (%)	*τ*_o_ (Pa)	*G*′ (Pa)	*G*″ (Pa)	tan δ
1	25	3	1	-	21.12	197	57.7	0.293
2	25	3	1	3% Eucalyptus essential oil	40.54	233	99.9	0.429
3	25	3	1	3% PVP	33.76	280	110	0.393
4	25	3	1	3% PEG	14.71	67.2	38.2	0.569
5	25	3	1	3% PEG + 3%PVP	37.89	274	138	0.504

**Table 2:** Delivery parameters for PU-based hydrogels loaded with 1% MX, determined by using different approaches.

Model		Parameter	Sample				
			1	2	3	4	5
Peppas and Sahlin [59]:		k_1_ (min^−*m*^)	0.02676 ±0.00145	0.06788 ±0.0011	0.20879 ±0.01063	0.17267 ±0.001841	0.09747 ±0.001541
$\frac{M_{t}}{M_{\infty }}=k_{1}\;t^{m}+k_{2}\;t^{2m}$	(5)	k_2_ (min^−2*m*^)	2.3621 × 10^−4^ ±2.5 × 10^−6^	1.47 × 10^−3^ ±2.37 × 10^−5^	0.01232 ±4.57 × 10^−4^	0.00967 ±2.23 × 10^−4^	0.00252 ±9.196 × 10^−4^
*m* = release exponent *k*_1_, *k*_2_ = release constants		*m*	0.72709 ±0.01379	0.58066 ±0.01379	0.53929 ±0.05349	0.42418 ±0.00322	0.44734 ±0.00311
		AIC	−103.21186	−84.45988	−59.7453	−67.2264	−69.64999
		R^2^	0.99851	0.99095	0.9590	0.96533	0.97844
Korsmeyer and Peppas [58]:		*k* (min^−*n*^)	0.03423 ±0.00206	0.08607 ±0.00387	0.32079 ±0.05616	0.21404 ±0.01706	0.11219 ±0.01210
$\frac{M_{t}}{M_{\infty }}=k\;t^{n}$	(6)	*n*	0.61064 ±0.01403	0.44614 ±0.01338	0.25511 ±0.00538	0.25596 ±0.003101	0.3577 ±0.00395
*n* = release exponent *k* = release constant		AIC	−85.43094	−69.5174	−51.9922	−54.3462	−55.82036
		R^2^	0.99547	0.98129	0.95261	0.9511	0.9624
Higuchi [63,64]:		*k*_H_ (min^−1/2^)	0.05836 ±0.00172	0.08514 ±0.00367	0.06823 ±0.01977	0.10193 ±0.00711	0.08301 ±0.00331
$\frac{M_{t}}{M_{\infty }}=k_{H}\;t^{1/2}$	(7)	AIC	−64.82541	−65.6559	−28.9514	−49.4438	−60.4066
*k_H_* = release constant		R^2^	0.98791	0.98002	0.82637	0.9478	0.9797
First order [65]:		*k*_11_ (min^−1^)	0.00938 ±2.093 × 10^−4^	0.01896 ±1.003 × 10^−3^	0.00425 ±1.0 × 10^−5^	0.04816 ±5.31 × 10^−4^	0.01798 ±4.096 × 10^−4^
$\ln\;\left(1-\frac{M_{t}}{M_{\infty }}\right)=-k_{11}\;t$	(8)	AIC	−71.70195	−53.1783	−23.7414	−47.399	−71.4366
*k*_11_ = release constant		R^2^	0.99405	0.9752	0.62313	0.92081	0.99187

*M_t_*, *M_∞_* = the amount of released drug at time *t* and at equilibrium, respectively; *M_t_/M_∞_* = the fraction of released drug at time *t*; *M_o_* = the initial amount of drug in the formulation; *t* = time.

## Data Availability

The original contributions presented in this study are included in the article. Further inquiries can be directed to the corresponding author.
